# Tribbles homolog 3 contributes to high glucose-induced injury in retinal pigment epithelial cells via binding to growth factor receptor-bound 2

**DOI:** 10.1080/21655979.2022.2056315

**Published:** 2022-04-23

**Authors:** Qin Liao, Xuefeng Gao

**Affiliations:** a Department of Ophthalmology, Chengdu Second People’s Hospital, Chengdu, china; b College of Management, Beijing Capital Normal University, Beijing

**Keywords:** Diabetic retinopathy, high glucose, GRB2, TRB3

## Abstract

Diabetic retinopathy (DR) is the most typical complication of diabetes, which severely threatens sight. Tribbles homolog 3 (TRB3), a kind of pseudokinase, is discovered to be highly expressed in diabetes and retinas after retinal detachment. TRB3 expression in human retinal pigment epithelial (hRPE) cells exposed to different concentrations of glucose was tested by RT-qPCR and western blot. Then, cells were induced with 30 mM high glucose (HG) to establish a DR cell model. Following TRB3 knockdown, cell viability estimation employed CCK-8 assay. The mRNA levels of inflammatory factors were detected by RT-qPCR. Reactive oxygen species (ROS) level was measured by DCFH-DA assay, and levels of oxidative stress markers were evaluated applying corresponding kits. Cell apoptosis was assayed by TUNEL assay and western blot. Following, the growth factor receptor-bound 2 (GRB2) expression was also examined by RT-qPCR and western blot. The interaction between TRB3 and GRB2 was verified by Co-IP assay. After GRB2 was overexpressed in HG-induced hRPE cells transfected with shRNA-TRB3, functional experiments were conducted again. The results manifested that TRB3 expression was elevated under HG conditions. Deficiency of TRB3 enhanced the viability while alleviated inflammation, oxidative stress, and apoptosis in HG-induced hRPE cells. GRB2 was also increased in HG-exposed hRPE cells. Moreover, GRB2 had a strong affinity with TRB3 and positively regulated by TRB3. After GRB2 overexpression, the effects of TRB3 knockdown on HG-stimulated hRPE cells were all reversed. Briefly, this study confirmed the promoting role of TRB3/GRB2 axis in the progression of DR.

## Highlights

TRB3 interacts with GRB2 in HG-triggered retinal pigment epithelial cells s

TRB3 modulates inflammation and oxidative stress in DR through binding to GRB2

TRB3 modulates cell damage in DR through binding to GRB2

## Introduction

Diabetic retinopathy (DR) is an ocular manifestation of diabetes, which often develops slowly and insidiously [1]. It remains the most prevalent cause of visual impairment and even irreversible blindness among adults on account of formation of new blood vessels, vitreous or anterior retinal hemorrhage [2,3]. As reported, DR has a tendency to occur in approximately 80% of diabetic patients with a 20-year disease course, and about 10% of DR patients are prone to inevitably develop blindness in spite of effective treatment [4–6]. The predominant risk factors for DR include diabetes duration, poor metabolic control, pregnancy, hypertension, hyperglycemia, poor control of blood lipids, kidney disease [7]. Despite the fact that great improvements in the treatment strategies of DR have been witnessed, more studies are still required to explore the etiology and pathogenesis of DR and develop promising new approaches to DR therapy [8].

TRB3, also named TRIB3, is a member of the Tribbles family of pseudokinases. A growing body of evidence has implied that TRB3 is a primary driving factor in diverse cellular functions, even if it possesses catalytically inactive kinase-like domain based on protein structure [9]. Recent reports have substantiated that TRB3 is essential for the development and progression of a variety of cancers. For instance, Hua et al. have presented that TRB3 stimulates the stemness property of colorectal cancer cells and boosts tumorigenesis [10]. Another study has proposed that TRB3 facilitates the aggressive phenotypes of lung adenocarcinoma cells [11]. Also, the finding that TRB3 influences the proliferation, migration, apoptosis, and chemotherapy resistance of hepatocellular carcinoma cells via mediating AKT signaling pathway is highlighted [12,13]. Notably, accumulating studies have revealed that TRB3 exhibits high expression in rats with diabetes [14,15]. Moreover, TRB3 confers insulin resistance in hepatocytes, which is one of the main pathological mechanisms of diabetes [16]. Further, TRB3 expression is increased in retinas after retinal detachment [17]. However, whether TRB3 participates in the process of DR needs to be explored.

GRB2 is a ubiquitously adaptor protein, which is implicated in numerous malignant tumors, such as multiple myeloma [18], lung cancer [19,20], hepatocellular carcinoma [21], esophageal squamous cell carcinoma [22], pancreatic cancer [23] and so on. These results implied that GRB2 chiefly serves as an oncogene. What is more, GRB2 is discovered to be overexpressed in the retina in a mouse model of DR [24]. Despite this, the correlation between TRB3 and GRB2 in DR remains unclear.

In the present study, we hypothesize that TRB3 may interact with GRB2 protein to be involved in the pathogenesis of DR and intend to carry out functional experiments and mechanism assays in a cell model of DR to validate this conjecture.

## Materials and methods

### Cell culture

hRPE cell line ARPE-19 was procured from the American Type Culture Collection (ATCC, USA) and Dulbecco’s modified Eagle’s medium (DMEM; Life Technologies, Carlsbad, USA) required for cell culture was maintained at 37°C with 5% CO_2_ with the addition of 10% fetal bovine serum (FBS; Gemini Bio) and 1% antibiotics (Sigma, Poznan, Poland). Cells were, respectively, treated with 5 mM glucose, 5 mM glucose, and 25 mM mannitol (MA) or 30 mM high glucose (HG) and divided into control group, MA group, and HG group successively.

### Cell transfection

The specific short hairpin RNAs (shRNAs) targeting TRB3 (shRNA-TRB3#1/2) and negative control (shRNA-NC) were offered by Shanghai Genechem. For up-regulation of GRB2, plasmids carrying GRB2 gene (Ov-GRB2) were designed by Sangon Biotech Co., Ltd, with Ov-NC as the empty vector. Plasmid transfection was performed in ARPE-19 cells with the adoption of Lipofectamine 3000 (Life Technologies). Cells were collected 48 h later for the following experiments [25].

### Reverse transcription-quantitative PCR (RT-qPCR)

Total RNA was obtained by lysing ARPE-19 cells with RNeasyMini kit (Qiagen), followed by the acquisition of cDNA through reverse transcription using ReverTra Ace qPCR RT kit (TOYOBO, Japan) in agreement with the manufacturer’s protocol. Then, ABI7500 Fast Real-time PCR system (ABI, Oyster Bay, NY) was employed for the execution of PCR analysis according to SYBR Green I Master Mix (Bioneer). The reaction system was conducted under the following conditions: 95°C for 10 min; followed by 40 cycles of 95°C for 10 sec and 60°C for 60 sec. The sequences were listed below: TRB3, forward, 5’- −3’, reverse, 5’- −3’; GRB2, forward, 5’- −3’, reverse, 5’- −3ʹRelative mRNA levels were measured with the aid of 2^−ΔΔCt^ method [26] and standardized by GAPDH. Primer sequences were as follows: TRB3 forward: 5’-TTTTCACAGACCCCGCCG-3’, reverse: 5’-ACTCCAACCGCTTCTTCCTG-3’; GRB2 forward: 5’-AAGCTACTGCAGACGACGAG-3’, reverse: 5’-GCCGCTGTTTGCTAAGCATT-3’; TNF-α forward: 5’- TCTCCCCTGGAAAGGACACC-3’, reverse 5’- GCAGGCAGAAGAGCGTGGT-3’; IL-1β forward: 5’- GAAATGATGGCTTATTACAGTGGC-3’, reverse 5’- GAAATGATGGCTTATTACAGTGGC-3’; IL-6 forward: 5’- ATGAGGAGACTTGCCTGGTGAA-3’, reverse 5’- GTTGGGTCAGGGGTGGTTATT-3’; GAPDH: forward: 5’- AATGGGCAGCCGTTAGGAAA −3’, reverse 5’- GCGCCCAATACGACCAAATC −3’.

### Cell counting Kit-8 (CCK-8) assay

ARPE-19 cells (5000 cells/well) inoculated in 96-well plates were cultivated in 10 μl CCK-8 solution (Haling Biotechnology, Shanghai, China) at established time points at 37°C. Cell viability was determined 2 h later under a microplate reader (BIOTEK, USA) by absorbance at 450 nm.

### Terminal-deoxynucleoitidyl transferase mediated nick end labeling (TUNEL)

With the application of TUNEL assay kit (BD Pharmingen, CA), the apoptotic rate was determined in compliance with the manufacturer’s protocol [27]. After being immobilized by 4% paraformaldehyde, ARPE-19 cells were permeated with 0.1% Triton X-100 and then subjected to TUNEL reagent (Clontech, Mountain View, CA). Nuclear staining was executed with DAPI (Haoran Biotechnology, Shanghai, China) for 10 min. At last, stained cells in five randomly selected areas were observed by a fluorescence microscope (Leica Microsystems GmbH).

### DCFH-DA assay

To detect ROS accumulation, ARPE-19 cells were washed with PBS in fresh DMEM. After the cell culture medium was removed, cells were incubated with 10 μΜ DCFH-DA (Jiancheng, Nanjing) at 37°C for half an hour in the dark. Finally, images were captured by a fluorescence microscope (Leica Microsystems GmbH), and ROS level was assessed with the aid of a flow cytometer (Merck, Darmstadt, Germany) [28].

### Detection of malondialdehyde (MDA) and superoxide dismutase (SOD)

MDA (A003-1-2) and SOD (A001-1-2) levels were measured by corresponding commercial kits (Jiancheng Biotech, Nanjing, China) according to the manufacturer’s guidance.

### Co-immunoprecipitation (Co-IP)

After rinsing with pre-cooled PBS, ARPE-19 cells were collected and lysed in pre-cooled cell lysis buffer (Beyotime Biotech Inc., China). After centrifugation at 13,000 x g for 10 min at 4°C, cell lysates were harvested and cultivated with 2 µg IgG antibody (cat. no. ab205718; 1:20; Abcam), TRB3 antibody (cat. no. ab75846; 1:10; Abcam) or GRB2 antibody (cat. no. ab32037; 1:50; Abcam) overnight at 4°C followed by 2 h precipitation with the addition of 0.2 mg protein A agarose beads (Thermo Fisher Scientific, Inc.) at room temperature. The immunoprecipitated protein complexes were collected and subjected to western blot detection after PBS washing [29].

### Western blot

Proteins were acquired by lysing ARPE-19 cells using RIPA buffer (Keygen, China), followed by protein concentration assay by a BCA protein assay kit (Pierce Chemical, USA). Proteins (30 µg per lane) were electrophoresed on 10% SDS-PAGE (Tanon Science & Technology Co., Ltd.), and placed on polyvinylidene fluoride membranes, which were subsequently impeded by 5% nonfat milk. Then, the membranes and primary antibodies were incubated together at 4°C overnight, followed by the cultivation with horseradish peroxidase-linked secondary antibody (cat. no. ab109489; 1:1000; Abcam) at room temperature. Protein bands were visualized by ECL system (Amersham Pharmacia Biotech) and analyzed by ImageJ software (v6; National Institutes of Health). The value was normalized to GAPDH. The primary antibodies used here including anti-TRB3 (cat. no. ab75846; 1:5000), anti-Bcl-2 (cat. no. ab32124; 1:1,000), anti-Bax (ab32503; 1:1000), anti-Cleaved caspase3 (cat. no. ab32042; 1:500), anti-caspase3 (ab32351; 1:5000), anti-Cleaved PARP (cat. no. ab32064; 1:1000), anti-PARP (cat. no. ab191217; 1:5000), anti-GRB2 (cat. no. ab32037; 1:5000) and anti-GAPDH (cat. no. ab9485; 1:2500) were all procured from Abcam.

### Statistical analyses

Experimental data were shown as the mean ± SD from three independent experiments. Statistical analyses were performed with the aid of SPSS 22.0 (Chicago, IL, USA) and GraphPad Prism 8.0 software (San Diego, CA, USA). Comparisons between the two groups were done by Student’s t-test. One-way ANOVA and Tukey’s post hoc test were applied when more than two groups were compared, with p < 0.05 seen as significant level. Each group was tested at least 3 times.

## Results

### TRB3 expression is increased in HG-treated hRPE cells in a concentration-dependent manner

**Figure 1.**
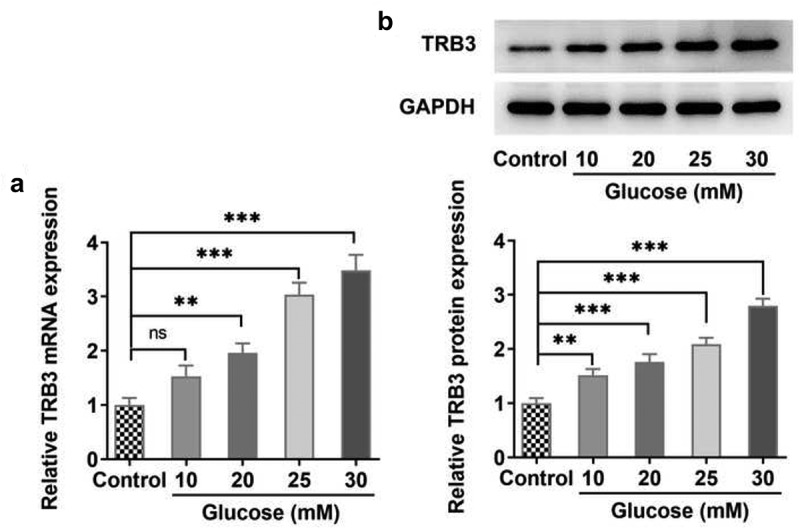
TRB3 expression is increased in HG-treated hRPE cells in a concentration-dependent manner. (a) RT-qPCR and (b) western blot tested TRB3 expression in hRPE cells exposed to different concentrations of HG. **P < 0.01, ***P < 0.001. TRB3, Tribbles homolog 3.

To verify the role of TRB3 in DR, hRPE cells were induced by different concentrations of HG (10, 20, 25, and 30 mM), and it was discovered that TRB3 expression was gradually elevated in HG-induced hRPE cells in a concentration-dependent manner, with the highest expression in hRPE cells exposed to 30 mM HG (Figure 1(a,b)). Therefore, 30 mM HG-induced hRPE cells were adopted to construct a DR cell model in the following experiments. These results revealed the concentration-dependent effect of HG on TRB3 expression in hRPE cells.

### TRB3 depletion alleviates HG-triggered hRPE cell impairment

**Figure 2.**
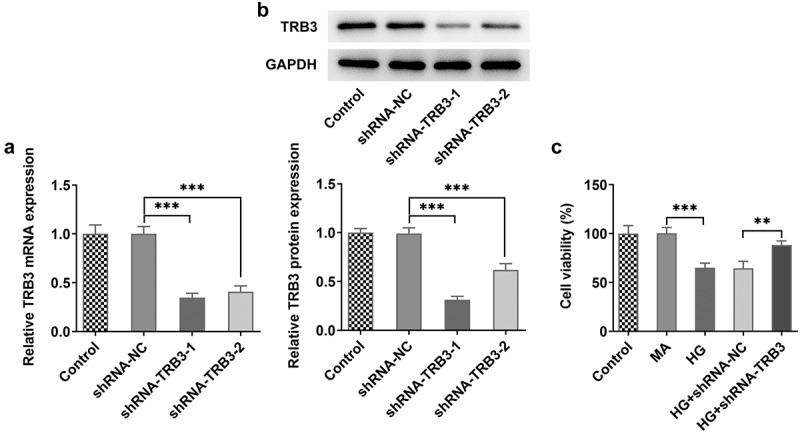
TRB3 depletion alleviates HG-triggered hRPE cell impairment. The transfection efficiency of shRNA-TRB3-1/2 plasmids was tested by (a) RT-qPCR and (b) western blot. (c) The impacts of TRB3 silencing on the viability of HG-treated hRPE cells were appraised by CCK-8 assay. **P < 0.01, ***P < 0.001. TRB3, Tribbles homolog 3. MA, mannitol. HG, high glucose.

In the subsequent functional experiments, RT-qPCR and western blot detected a successful decline in TRB3 expression in hRPE cells after transfection of shRNA-TRB3-1/2 plasmids (Figure 2(a,b)). shRNA-TRB3-1 was selected for the following experiments for its better knockdown efficacy. It was observed that the viability of hRPE cells was notably reduced under HG conditions relative to the MA group via CCK-8 assay. Moreover, knockdown of TRB3 improved the viability of HG-mediated hRPE cells compared to the HG + shRNA-NC group (Figure 2(c)). To sum up, deficiency of TRB3 enhanced the viability of hRPE cells exposed to HG.

### TRB3 inhibition protects HG-insulted hRPE cells against inflammation and oxidative stress

**Figure 3.**
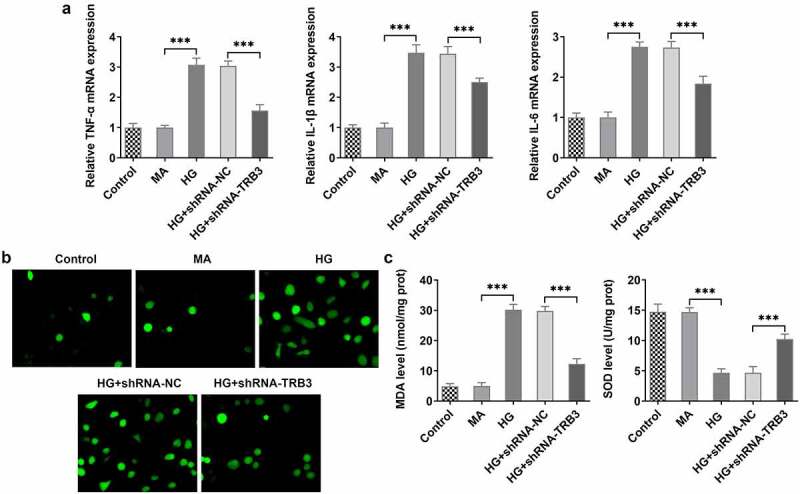
TRB3 inhibition protects HG-insulted hRPE cells against inflammation and oxidative stress. (a) TNF-α, IL-1β and IL-6 levels were determined by RT-qPCR. (b) ROS accumulation was detected by DCFH-DA assay. (c) MDA and SOD levels were confirmed by corresponding kits. ***P < 0.001. TRB3, Tribbles homolog 3. MA, mannitol. HG, high glucose. TNF-α, tumor necrosis factor alpha. IL-1β, interleukin-1beta. IL-6, interleukin-6. MDA, malondialdehyde. SOD, superoxidase dismutase.

Inflammatory response and oxidative stress are commonly acknowledged as primary contributors of DR [30,31]. Based on RT-qPCR analysis, it was noticed that the expressions of inflammatory factors including TNF-α, IL-1β, and IL-6 were all on the rise in hRPE cells after exposure to HG while distinctly reduced after TRB3 was depleted (Figure 3(a)). ROS accumulation, which is known as a leading cause of oxidative stress, was investigated by DCFH-DA assay. The results implied that the production of ROS was boosted in hRPE cells in response to HG induction, while the shortage of TRB3 hampered ROS formation (Figure 3(b)). In addition, MDA level was elevated while SOD level was declined in HG-treated hRPE cells and silencing of TRB3 led to the decrease in MDA level and an increase in SOD level (Figure 3(c)). Overall, TRB3 knockdown reduced HG-elicited inflammation and oxidative stress in hRPE cells.

### TRB3 interference impedes HG-evoked apoptosis in hRPE cells

**Figure 4.**
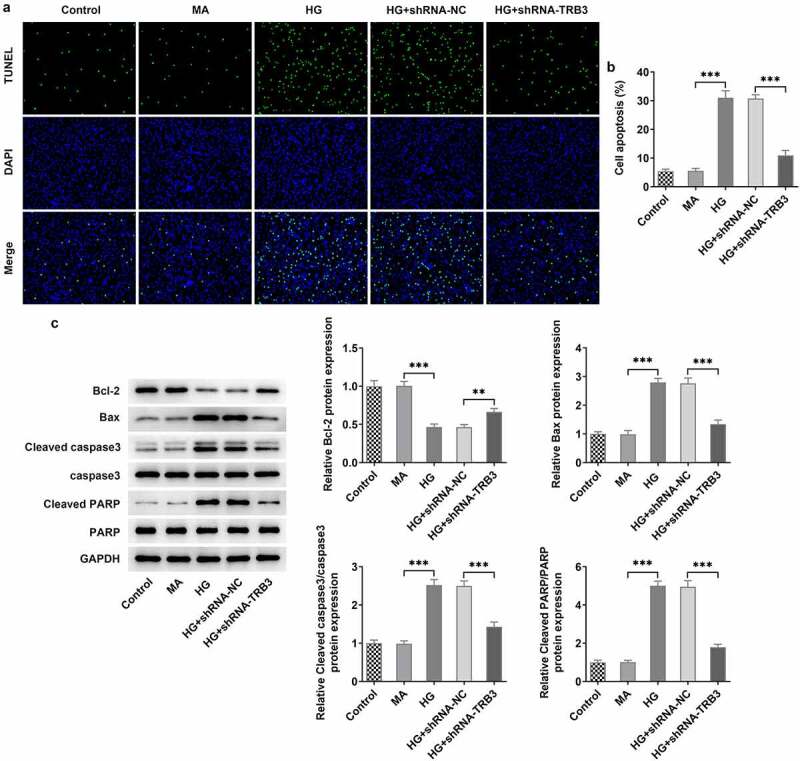
TRB3 interference impedes HG-evoked apoptosis in hRPE cells. (a) TUNEL assay estimated the influence of TRB3 depletion on the apoptosis of HG-stimulated hRPE cells and (b) the quantification. (c) The protein levels of apoptosis-related factors were analyzed by western blot. **P < 0.01, ***P < 0.001. TRB3, Tribbles homolog 3. MA, mannitol. HG, high glucose. Bcl-2, B cell lymphoma-2. Bax, BCL-2 associated X.

At the same time, the results from TUNEL assay revealed that HG-aggravated apoptosis of hRPE cells was alleviated when TRB3 was down-regulated (Figure 4(a,b)). Similar results could also be seen in western blot analysis, as evidenced by the outcome that the lessened Bcl-2 protein level and the reinforced Bax, cleaved caspase3/caspase 3, and cleaved PARP/PARP protein levels in HG-insulted hRPE cells were all restored by TRB3 insufficiency (Figure 4(c)). In summary, silencing of TRB3 played an anti-apoptotic role in HG-treated hRPE cells.

### TRB3 interacts with GRB2 in hRPE cells under HG conditions

**Figure 5.**
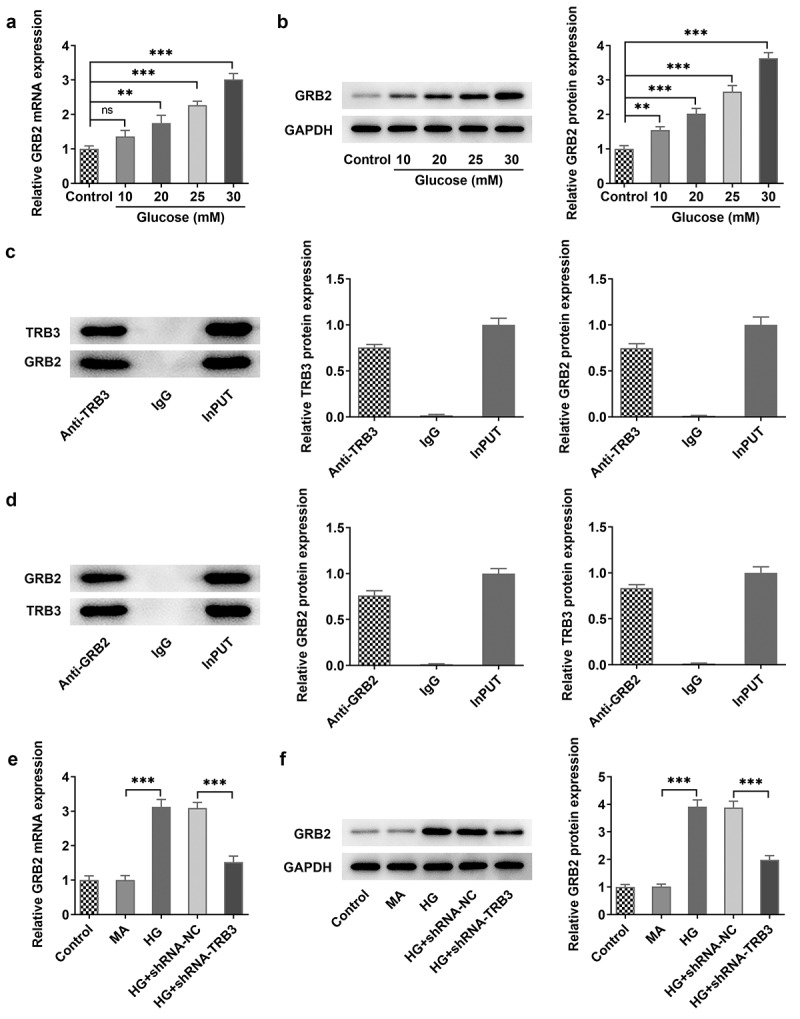
TRB3 interacts with GRB2 in hRPE cells under HG conditions. (a) RT-qPCR and (b) western blot tested GRB2 expression in hRPE cells exposed to different concentrations of HG. (c-d) Co-IP assay testified the binding to TRB3 to GRB2. (e) RT-qPCR and (f) western blot tested GRB2 expression when TRB3 was down-regulated. **P < 0.01, ***P < 0.001. n.s., not significant. TRB3, Tribbles homolog 3. MA, mannitol. HG, high glucose. GRB2, growth factor receptor-bound 2.

Unexpectedly, TRB3 was predicted to bind to GRB2 according to the Biogrid database (https://thebiogrid.org/). Moreover, RT-qPCR and western blot analysis also indicated that HG concentration-dependently elevated GRB2 expression in hRPE cells and GRB2 displayed the highest expression in hRPE cells when exposed to 30 mM HG (Figure 5(a,b)). Thereafter, the direct binding of TRB3 to GRB2 was verified by Co-IP assay (Figure 5(c,d)). Furthermore, we found that transfection of shRNA-TRB3 significantly cut down the mRNA and protein level of GRB2, which suggested a positive correlation between TRB3 and GRB2 (Figure 5(e,f)). Overall, TRB3 could bind to GRB2 in HG-induced hRPE cells.

### TRB3 modulates HG-triggered hRPE cell damage through binding to GRB2

**Figure 6.**
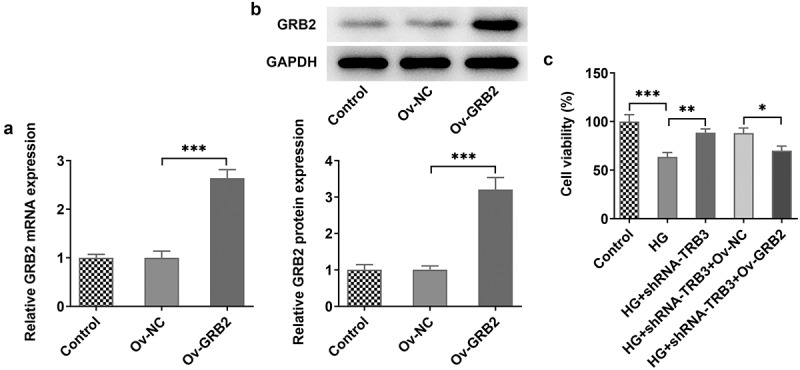
TRB3 modulates HG-triggered hRPE cell damage through binding to GRB2. The transfection efficiency of Ov-GRB2 plasmid was tested by (a) RT-qPCR and (b) western blot. (c) CCK-8 assay evaluated cell viability. *P < 0.05, **P < 0.01, ***P < 0.001. TRB3, Tribbles homolog 3. MA, mannitol. HG, high glucose. GRB2, growth factor receptor-bound 2.

To testify that TRB3 might affect cell viability via binding to GRB2, we transfected Ov-GRB2 into hRPE cells and found that GRB2 was up-regulated by RT-qPCR and western blot analysis (Figure 6(a,b)). Additionally, CCK-8 assay demonstrated that the viability of HG-exposed hRPE cells inhibited by TRB3 was enhanced by GRB2 elevation (Figure 6(c)). Briefly, TRB3 elevated the viability of hRPE cells upon exposure to HG depending on their interaction with GRB2.

### TRB3 has impacts on HG-provoked inflammation and oxidative stress in hRPE cells via interaction with GRB2

**Figure 7.**
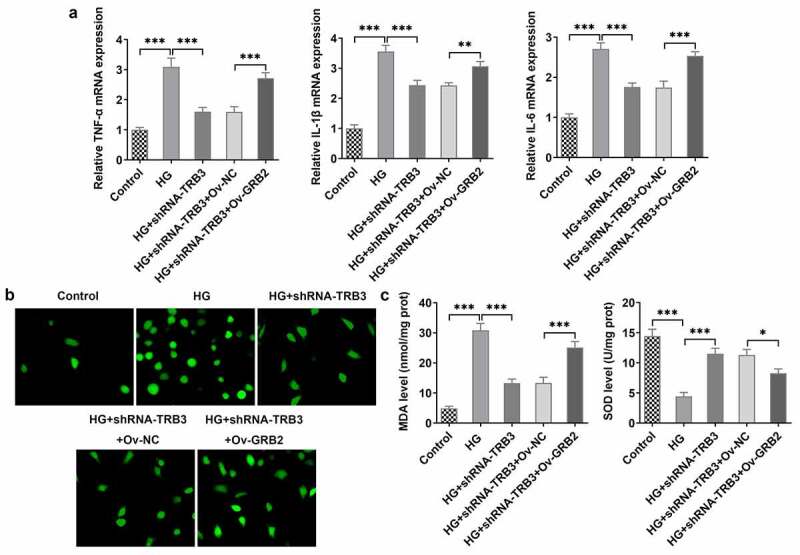
TRB3 has impacts on HG-provoked inflammation and oxidative stress in hRPE cells via interaction with GRB2. (a) TNF-α, IL-1β and IL-6 levels were determined by RT-qPCR. (b) ROS accumulation was detected by DCFH-DA assay. (c) MDA and SOD levels were confirmed by corresponding kits. *P < 0.05, **P < 0.01, ***P < 0.001. TRB3, Tribbles homolog 3. MA, mannitol. HG, high glucose. GRB2, growth factor receptor-bound 2. TNF-α, tumor necrosis factor alpha. IL-1β, interleukin-1beta. IL-6, interleukin-6. MDA, malondialdehyde. SOD, superoxidase dismutase.

At the same time, RT-qPCR analysis uncovered that the expressions of TNF-α, IL-1β, and IL-6, which were declined by TRB3 knockdown, was in turn elevated by overexpression of GRB2 in HG-exposed hRPE cells (Figure 7(a)). Further detection of ROS generation hinted that after GRB2 was overexpressed, the lessened ROS activity in HG-treated hRPE cells transfected with shRNA-TRB3 was increased (Figure 7(b)). As expected, TRB3 depletion down-regulated MDA level, while up-regulated SOD level in HG-insulted hRPE cells, while this result was offset by GRB2 elevation (Figure 7(c)). Taken together, TRB3 down-regulation eased HG-triggered inflammation and oxidative stress in hRPE cells via GRB2 suppression.

### TRB3 affects HG-mediated hRPE cell apoptosis by binding with GRB2

**Figure 8.**
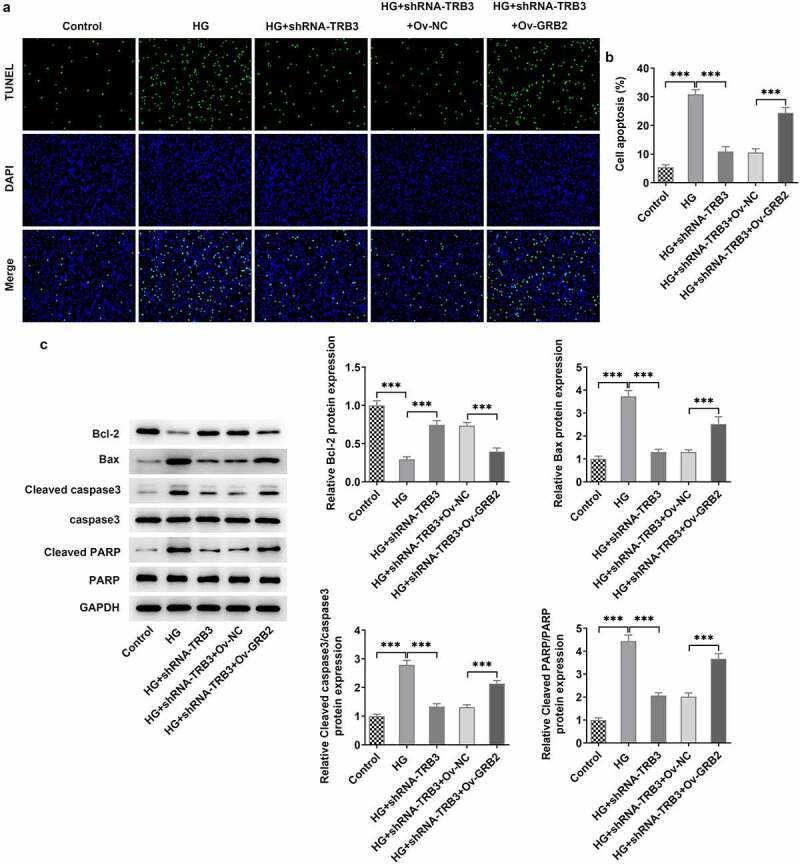
TRB3 affects HG-mediated hRPE cell apoptosis by binding with GRB2. (a) TUNEL assay estimated the apoptosis of HG-stimulated hRPE cells and (b) the quantification. (c) The protein levels of apoptosis-related factors were analyzed by western blot. ***P < 0.001. TRB3, Tribbles homolog 3. MA, mannitol. HG, high glucose. GRB2, growth factor receptor-bound 2. Bcl-2, B cell lymphoma-2. Bax, BCL-2 associated X.

In the same way, TUNEL assay clarified that the strengthened apoptotic ability of hRPE cells in response to HG was weakened by TRB3 reduction while was elevated again when GRB2 was up-regulated (Figure 8(a,b)). As reflected by western blot analysis, the enhanced Bcl-2 protein level and the falling Bax, cleaved caspase3/caspase 3, and cleaved PARP/PARP protein levels imposed by TRB3 deficiency were all counteracted by GRB2 up-regulation (Figure 8(c)). Collectively, TRB3 interacted with GRB2 to modulate HG-elicited apoptosis in hRPE cells.

## Discussion

DR is a metabolic and multifactorial disease whose pathogenesis is extremely complicated [32]. Despite the low incidence rate of DR in China, it poses great economic burden on families and society and brings great impact to human health [33]. As is well known, hyperglycemia represents a pathological hallmark of DR [34]. In the continuous stimulation of HG environment, cell–cell communication is blocked, and retinal homeostasis is disrupted [35]. As a consequence, metabolic and physiologic abnormalities in the retina including inflammation and oxidative stress, which are the typical pathological features of DR may occur, thereby leading to the impairment of blood retinal barrier [36–39]. For instance, the viability of rat retinal capillary endothelial cells is reduced under 30 mM glucose conditions [40]. In addition, 30 mM glucose exposure yields a tremendous increase on the apoptosis and oxidative stress of retinal endothelial cells [41]. And because RPE cells are a component of a blood retinal barrier whose dysfunction has been reported to be engaged in the pathological progress of DR [42]. Thence, 30 mM HG was used to induce ARPE-19 cells to construct a DR cell model in the current study.

To the best of our knowledge, TRB3 has been emphasized as a controversial apoptosis-regulated gene for its both pro-apoptotic and anti-apoptotic roles. For example, Cao et al. have shown that TRB3 drives cell proliferation and migration while eases cell apoptosis in lung adenocarcinoma [11]. Cheng et al. have proved that interference of TRB3 suppresses cardiomyocyte apoptosis under hypoxia [43]. To date, it is well documented that TRB3 plasma level is up-regulated in diabetic patients [44]. TRB3 is widely expressed in retinas after retinal detachment [17]. It was revealed from our data that TRB3 expression was increased in hRPE cells exposed to HG in a concentration-dependent manner. Moreover, the results from loss-of-function experiments manifested that HG treatment observably weakened the viability of hRPE cells. TRB3 has been reported to increase glucose intolerance and regulate cell proliferation [45]. Under this condition, interference of TRB3 significantly aggravated cell viability.

In recent years, inflammatory response involved in DR has attracted much attention [46,47]. TNF-α is one of the most pivotal inflammatory cytokines and regarded as a candidate biomarker for DR [48]. Also, IL-1β and IL-6 play a fundamental role in inflammation [49,50]. What is more, IL-1β and IL-6 are abundant in DR and related to the severity of DR [51,52]. Through our investigation, it was discovered that TNF-α, IL-1β, and IL-6 expression were all ascending after induced by HG and down-regulation of TRB3 declined their expression. Additionally, oxidative stress is well accepted as a predisposing driver of DR and is reflected by excess accumulation and/or impaired removal of ROS [31]. SOD, a principle antioxidant enzyme, eliminates ROS to alleviate oxidative damage [53]. By contrast, MDA is one of the most frequently measured biomarkers of oxidative stress [54]. In the same way, the increased ROS, MDA, and the reduced SOD levels in HG-insulted hRPE cells were all reversed by depletion of TRB3. In our experiments, we found that HG condition stimulated the apoptosis of hRPE cells and the apoptotic ability was weakened when TRB3 was down-regulated.

As reported, GRB2 binds to receptor tyrosine kinases and cytokine receptors and interacts with downstream proteins [55]. At the same time, GRB2 is conventionally known to play oncogenic roles in cancers [56]. Based on Biogrid database, it was predicted that TRB3 might interact with GRB2. More importantly, GRB2 displayed a higher expression in hRPE cells exposed to HG in a concentration-dependent manner. In accordance with our findings, a previous study has illustrated that GRB2 is overexpressed in DR tissues [24]. The binding of TRB3 to GRB2 was also verified by Co-IP assay. Besides, GRB2 was positively modulated by TRB3. The findings above testified this speculation. The succeeding parts of the study further confirmed that after GRB2 was overexpressed, the elevated viability, the lessened TNF-α, IL-1β, and IL-6 expression, the declined ROS, MDA levels, and apoptosis and the increased SOD levels in HG-treated hRPE cells owing to TRB3 deficiency were all restored, which indicated that TRB3 contributed to HG-stimulated hRPE cell injury through interaction with GRB2.

## Conclusion

In summary, this study provided the first evidence demonstrating that TRB3 knockdown suppressed cell viability and triggered inflammation, oxidative stress, and apoptosis in DR by binding to GRB2, which set forth a novel molecular mechanism of TRB3 in DR and identified TRB3 as a new therapeutic target for DR therapy from bench to clinic.

## Data Availability

The analyzed data sets generated during the present study are available from the corresponding author on reasonable request.
